# Macrolide Resistance in MORDOR I: A Cluster-Randomized Trial in Niger

**DOI:** 10.1056/NEJMc1901535

**Published:** 2019-06-06

**Authors:** Thuy Doan, Ahmed M Arzika, Armin Hinterwirth, Ramatou Maliki, Lina Zhong, Susie Cummings, Samarpita Sarkar, Cindi Chen, Travis C Porco, Jeremy D Keenan, Thomas M Lietman, for the MORDOR Study Group

**Affiliations:** 1 Francis I. Proctor Foundation; 2 The Carter Center, Niger

Mass drug administration with azithromycin reduced childhood mortality in MORDOR I and II in Niger, although antibiotic resistance remains a major concern.^1-3^ MORDOR I was a trial that did not itself include morbidity assessments.1 However, 30 communities in Niger were randomly selected from the same pool as the mortality trial, and randomly assigned to either azithromycin or placebo distributed biannually to pre-school children as in MORDOR I (see protocol and SAP at nejm.org for full study details). The mean (±SD) placebo and azithromycin coverage over the four twice-yearly distributions was 82 ± 6% and 79 ± 8%, respectively. Ethical approval was obtained from the University of California San Francisco Committee for Human Research and the Ethical Committee of the Niger Ministry of Health. We obtained oral consents from guardians prior to treatment and swab collection. No incentives were offered. All analyses were at the community level.

Here, we compare the proportion of macrolide-resistant pneumococcus in pre-school children between azithromycin and placebo-treated communities, using broth dilution assays on pneumococcus isolated from nasopharyngeal swabs collected at 24 months (approximately 6 months after the fourth biannual treatment). Pneumococcus isolation and resistance testing were performed according to standard protocols at ARUP, a CLIA-certified reference laboratory, using breakpoints as documented in the Clinical and Laboratory Standard Institute guidelines (see Supplementary Appendix). For this study, intermediate and resistant cases were considered as resistant. Because the gut is a reservoir for antibiotic resistance genes, we also evaluated the resistome from rectal samples at 24 months using metagenomic DNA sequencing and compared the non-host sequences against a curated antibiotic resistance database.^4,5^

At 24 months, the proportion of macrolide resistance in nasopharyngeal *S. pneumoniae* at the community level was higher in the azithromycin-treated communities (mean 12.3%, 95% confidence interval 5.7—20.0%) than in the placebo-treated communities (2.9%, 0—6.1%, *p*=0.02, see Table 1 and Table S1 in the Supplementary Appendix). Similarly, macrolide resistance determinants in the gut were more prevalent in the azithromycin-treated communities (68.1%, 60.4—74.8% vs 46.3%, 35.9—52.9%; *p* <0.001). We next evaluated whether mass oral azithromycin administration was associated with an increase in resistance to other antibiotics. The proportion of isolated pneumococcus resistant to penicillin was similar between treatment arms: 18.7%, (8.2%—30.6%) in the azithromycin group versus 22.3% (10.2%—37.8%) in the placebo group (*p*=0.72). As with the nasopharyngeal samples, we found no evidence of a difference between arms for the rectal samples in the non-macrolide classes (Table 1 and Table S2 in the Supplementary Appendix).

**Table 1:** Nasopharyngeal pneumococcal and gut antibiotic resistance of pre-school children at 24 months.

Antibiotic	Mean Proportion, % (95% Confidence Interval) *Streptococcus pneumoniae* resistance (phenotypic)	
	Placebo	Azithromycin
**Erythromycin**	**2.9 (0 to 6.1)**	**12.3 (5.7 to 20.0)**
Clindamycin	1.7 (0 to 4.3)	9.0 (4.3 to 14.1)
Penicillin	22.3 (10.2 to 37.8)	18.7 (8.2 to 30.6)
TMP-SMX	77.1 (65.4 to 88.1)	84.7 (76.4 to 92.4)
Doxycycline	50.1 (33.7 to 66.0)	60.1 (50.8 to 70.5)
Linezolid	0 (0 to 2.3)	0 (0 to 2.3)
Ceftriaxone	0 (0 to 2.3)	0 (0 to 2.3)
Vancomycin	0 (0 to 2.3)	0 (0 to 2.3)
Levofloxacin	0 (0 to 2.3)	0 (0 to 2.3)
Meropenem	0 (0 to 2.3)	0 (0 to 2.3)
Antibiotic	Mean Prevalence, % (95% Confidence Interval) **Genetic resistance determinants in stool**	
	Placebo	Azithromycin
**Macrolides**	**46.3 (35.9 to 52.9)**	**68.1 (60.4 to 74.8)**
Aminocoumarins	5.5 (2.9 to 8.6)	9.2 (5.2 to 13.1)
Aminoglycosides	30.9 (25.2 to 37.8)	37.6 (30.4 to 45.3)
Bacitracin	17.5 (10.8 to 26.1)	17.7 (12.8 to 25.9)
β-lactam	64.0 (57.5 to 72.9)	67.6 (59.7 to 74.5)
Cationic	33.2 (25.5 to 41.8)	35.2 (29.3 to 42.0)
Elfamycins	47.0 (39.4 to 54.0)	48.0 (36.0 to 54.7)
Fluoroquinolones	28.3 (20.9 to 36.7)	27.4 (20.2 to 35.2)
Fosfomycin	0 (0 to 2.3)	0.6 (0 to 1.8)
Glycopeptides	1.2 (0 to 3.0)	1.3 (0 to 3.2)
Metronidazole	21.9 (15.5 to 28.5)	31.8 (21.7 to 41.3)
Multi-drug-resistance	44.9 (36.6 to 54.4)	43.6 (37.9 to 50.3)
Phenicol	4.4 (1.9 to 8.0)	5.6 (1.9 to 13.9)
Rifampin	13.8 (9.2 to 19.5)	16.8 (10.8 to 24.7)
Sulfonamide	23.2 (17.0 to 30.4)	16.7 (9.9 to 26.8)
Tetracycline	74.0 (68.5 to 79.6)	75.4 (68.7 to 81.0)
Trimethoprim	49.5 (40.2 to 58.2)	50.8 (43.1 to 59.1)

In conclusion, targeted biannual mass oral azithromycin to pre-school children in Niger increases resistance to macrolides (and related clindamycin resistance), but we found no evidence of increased resistance to other classes of antibiotics at 2 years. This is consistent with results from trachoma programs, which typically distribute annually in ages 6 months through adults. The longer-term effects of prolonged mass azithromycin distributions to pre-school children remain to be determined.^3^ Any policy for implementation of mass antibiotic administration should be coupled with careful monitoring for antibiotic resistance.

**Trial Registration.** Clinicaltrials.gov NCT02047981
