# Supplementary material for: Macrolide Resistance in MORDOR I: A Cluster-Randomized Trial in Niger
Source: N Engl J Med. Author manuscript; Available in PMC 2019 Jun 6. (PMC6518950; doi:10.1056/NEJMc1901535)
Supplement: Supplementary file 1 [file NEJM-2019-1901535-s1.pdf]

APPENDIX

Table of Contents

Research Team…………………………………………………………….2
Acknowledgements……………………………………………………......2
Laboratory Procedures………………………………………………….3
Statistical Analysis……………………………………………………...4
Table S1…………………………………………………………………...5
Table S2…………………………………………………………………...6

Research Team

The following investigators participated in MORDOR: *University of California, San Francisco, San Francisco, CA, USA* – Catherine A Cook, Sun Y Cotter, Thuy Doan, Dionna M Fry, Bruce D Gaynor, Jeremy D Keenan, Elodie Lebas, Ying Lin, Thomas M Lietman, Kieran S O'Brien, Catherine E Oldenburg, Travis C Porco, Kathryn J Ray, Philip J Rosenthal, George W Rutherford, Nicole E Stoller, Benjamin Vanderschelden, John P Whitcher, Zhaoxia Zhou, Lina Zhong; *London School of Hygiene and Tropical Medicine, London, UK* – Robin L Bailey, Sarah E Burr, John Hart, David CW Mabey, Anthony W Solomon; *Johns Hopkins University, Baltimore, MD, USA* – Evan M Bloch, Christian L Coles, Kurt Dreger, Hemjot Kaur, Alain B Labrique, Beatriz Munoz, Alfred Sommer, Jerusha Weaver, Sheila K West; *Blantyre Institute for Community Ophthalmology, Blantyre, Malawi* – Alvin Chisambi, Khumbo Kalua, Zachariah Kamwendo; *University of Malawi College of Medicine, Blantyre, Malawi* – Ken Maleta; *The Carter Center, Atlanta, GA, USA* – E Kelly Callahan, Aisha E Stewart; *The Carter Center Niger, Niamey, Niger* – Ahmed M Arzika, Abdou Moumouni Goundara, Salissou Kane, Ramatou Maliki; *Programme National de Santé Oculaire, Niamey, Niger* – Amza Abdou, Nassirou Beido, Boubacar Kadri; *Muhimbili University of Health and Allied Sciences, Dar es Salaam, Tanzania* – Mabula Kasubi; *National Institute for Medical Research, Dar es Salaam, Tanzania* – Leonard Mboera, Zakayo Mrango; *International Trachoma Initiative, Decatur, GA, USA*- Paul M Emerson, Huub Gelderbloom, MD, PJ Hooper.

The steering committee for the trial consisted of the following investigators: Robin L Bailey, Jeremy D Keenan, Thomas M Lietman (PI), Travis C Porco, and Sheila K West.

Acknowledgements

We thank the program officers from the trial's sponsor: *Bill & Melinda Gates Foundation, Seattle, WA, USA*– Rasa Izadnegahdar, Julie Jacobson, Thomas Kanyok, Erin Shutes, Chris Damman, and Supriya Kumar. We also thank the members of the Data and Safety Monitoring Committee: *University of Washington, Seattle, WA, USA* – Judd L Walson; *Liverpool School of Tropical Medicine, Liverpool, UK* – Allen W Hightower; *Loyola University, Chicago, IL, USA* – Emily E Anderson, *Berhan Public Health & Eye Care Consultancy, Addis Ababa, Ethiopia* – Wondu Alemayehu; *Tulane University, New Orleans, LA, USA* – Latha Rajan. We thank the laboratory personnel at ARUP, *Salt Lake City, USA* – Kimberly Hanson, Heidi Romero. Finally, we would like to thank MIBAC, and the staff at Biblioteca Angelica.

Laboratory Procedures

Nasopharyngeal Samples: 10 samples per village (300 total) at the 24-month timepoint were randomly chosen for antibiotic resistance testing. Pneumococcus isolation and resistance testing were performed according to standard protocols at ARUP, a CLIA-certified laboratory and a non-profit enterprise of the University of Utah. Briefly, swabs were inoculated onto agar containing 5% sheep blood and 5 mg/L of gentamycin, then incubated at 37°C in 5% CO_2 for 18-24 hours. Pneumococcus was identified by alpha-hemolysis and the results of 3 phenotypic/biochemical tests (optochin sensitivity, inulin fermentation, and bile solubility). Broth dilution assays were performed to determine antibiotic susceptibilities for erythromycin, clindamycin, doxycycline, meropenem, levofloxacin, penicillin, ceftriaxone, trimethoprim-sulfamethoxazole, linezolid, and vancomycin using the breakpoints as documented in the Clinical and Laboratory Standard Institute guidelines M100 ED28:2018.

MIC Breakpoints for *Streptococcus pneumoniae**

	**Interpretive Categories and MIC Breakpoints µg/mL**		
**Antimicrobial Agent**	S	I	R
Penicillin (nonmeningitis)	<=2	4	>=8
Penicillin (meningitis)	<=0.06	-	>=0.12
Penicillin (oral)	<=0.06	0.12-1	>=2
Erythromycin	<=0.25	0.5	>=1
Clindamycin	<=0.25	0.5	>=1
Meropenem	<=0.25	0.5	>=1
Ceftriaxone (meningitis)	<=0.5	1	>=2
Ceftriaxone (nonmeningitis)	<=1	2	>=4
Vancomycin	<=1	-	
Levofloxacin	<=2	4	>=8
Trimethoprim/sulfa	<=0.5/9.5	1/19-2/38	>=4/76
Linezolid	<=2	-	
Doxycyline	<=0.25	0.5	>=1

*http://em100.edaptivedocs.info/GetDoc.aspx?doc=CLSI%20M100%20ED28:2018&sbssok=CLSI%20M100%20ED28:2018%20TABLE%202G&format=HTML&hl=MIC%20breakpoints

Rectal Samples: DNA was extracted from the 600 fecal samples (10 samples per village, 300 samples at baseline and 300 samples at 24 months) using the Norgen stool DNA isolation kits (Norgen, Ontario, Canada) per manufacturer's instructions. Double-stranded DNA was fragmented, size selected, and converted to Illumina libraries using the NEBNext Ultra II DNA Library Prep Kit (E7645) according to the manufacturer's recommendation and then amplified with 11 PCR cycles. Samples were sequenced on the Illumina HiSeq 4000 or Novaseq using 150-nucleotide (nt) paired-end sequencing. An initial human-sequence removal step was accomplished as previously described.[4] Another round of human reads removal was performed using the very-sensitive-local mode of Bowtie2 (v2.2.4) with the same hg38 and panTro4 reference genomes. The remaining non-host read pairs were then aligned to the MEGARes reference antimicrobial database using BWA with default settings.[5] Only antibiotic resistance determinants (ARDs) with gene fraction of >80% were identified as present in the sample and included for further analyses. Each identified ARD was classified at the class and mechanism level. Macrolide resistance determinants belong to the macrolide-lincosamide-streptogramin

(MLS) class. Those resistance mechanisms include macrolide resistance efflux pumps, macrolide phosphotransferases, erm 23S rRNA methyltransferases, and cfr 23S rRNA methyltransferases.[5] A sample was determined to be macrolide resistant even if 1 ARD in the MLS class was detected.

Statistical Analysis

Phenotypic intermediate and resistant cases were considered drug-resistant for the purpose of this report. We calculated the proportion of isolates exhibiting erythromycin resistance for each community as the number exhibiting erythromycin resistance divided by the total number of isolates identified. We reported the average of these across communities in each arm (omitting communities for which no specimens yielded testable isolates). Comparison was conducted between the two treatment arms using the t-statistic, with significance testing performed by Monte Carlo permutation (N=10,000). Similarly, we compared the proportion of resistant isolates for the other drugs tested, as well as the overall proportion of isolates that grew. Bootstrapped 95% confidence intervals for the proportion of resistant isolates were reported provided at least one resistant isolate was found, and binomial upper 97.5% confidence limits were reported when no resistant isolates were found. Permutation tests and bootstrap analyses were conducted at the community level to account for the cluster-randomized design. Analysis of antibiotic resistance determinants for the rectal samples was conducted using a linear model for the prevalence estimated for each community, adjusted for baseline (ANCOVA). All analyses were conducted using the R package v. 3.5.0 for MacIntosh (R Foundation for Statistical Computing, Vienna, Austria).

Table S1: Macrolide resistance in nasopharyngeal pneumococci isolated from pre-school children at 24 months.

Village	*Arm*	*Erythromycin Resistant (N)*	*Clindamycin Resistant (N)*	*Total Samples Grew Pneumococcus (N)*
1	Placebo	0	0	0
2	Placebo	0	0	10
3	Placebo	0	0	4
4	Placebo	0	0	1
7	Placebo	0	0	6
8	Placebo	1	1	9
9	Placebo	1	0	6
16	Placebo	0	0	9
17	Placebo	0	0	5
18	Placebo	0	0	7
19	Placebo	0	0	3
20	Placebo	0	0	8
21	Placebo	0	0	1
22	Placebo	0	0	5
25	Placebo	1	1	8
5	Azithromycin	1	0	6
6	Azithromycin	0	0	5
10	Azithromycin	2	2	9
11	Azithromycin	0	0	0
12	Azithromycin	0	0	8
13	Azithromycin	0	0	2
14	Azithromycin	1	1	8
15	Azithromycin	2	1	4
23	Azithromycin	0	0	3
24	Azithromycin	1	1	7
26	Azithromycin	1	1	8
27	Azithromycin	0	0	0
28	Azithromycin	0	0	7
29	Azithromycin	1	1	5
30	Azithromycin	1	1	9

Table S2: ***P*-values for comparing antibiotic resistance between arms from stool**

Antibiotic Class	**ANCOVA *P* -value**
**Macrolides**	**0.0009**
Aminocoumarins	0.12
Aminoglycosides	0.23
Bacitracin	0.89
β-lactam	0.67
Cationic	0.53
Elfamycins	0.91
Fluoroquinolones	0.95
Fosfomycin	0.35
Glycopeptides	0.98
Metronidazole	0.12
Multi-drug-resistance	0.60
Phenicol	0.67
Rifampin	0.46
Sulfonamide	0.28
Tetracycline	0.83
Trimethoprim	0.78
